# Autoantigen Treatment in Type 1 Diabetes: Unsolved Questions on How to Select Autoantigen and Administration Route

**DOI:** 10.3390/ijms21051598

**Published:** 2020-02-26

**Authors:** Johnny Ludvigsson

**Affiliations:** Crown Princess Victoria Children’s Hospital and Div of Pediatrics, Dept of Biomedical and Clinical Sciences, Lnköping university, SE 58185 Linköping, Sweden; Johnny.Ludvigsson@liu.se

**Keywords:** type 1 diabetes, autoantigen treatment, oral administration, combination therapy, GAD-alum, vitamin D, intralymphatic treatment

## Abstract

Autoantigen treatment has been tried for the prevention of type 1 diabetes (T1D) and to preserve residual beta-cell function in patients with a recent onset of the disease. In experimental animal models, efficacy was good, but was insufficient in human subjects. Besides the possible minor efficacy of peroral insulin in high-risk individuals to prevent T1D, autoantigen prevention trials have failed. Other studies on autoantigen prevention and intervention at diagnosis are ongoing. One problem is to select autoantigen/s; others are dose and route. Oral administration may be improved by using different vehicles. Proinsulin peptide therapy in patients with T1D has shown possible minor efficacy. In patients with newly diagnosed T1D, subcutaneous injection of glutamic acid decarboxylase (GAD) bound to alum hydroxide (GAD-alum) can likely preserve beta-cell function, but the therapeutic effect needs to be improved. Intra-lymphatic administration may be a better alternative than subcutaneous administration, and combination therapy might improve efficacy. This review elucidates some actual problems of autoantigen therapy in the prevention and/or early intervention of type 1 diabetes.

## 1. Introduction

For almost 100 years, patients with type 1 diabetes mellitus (T1D) have been given “palliative” treatment with insulin [1]. Many patients have a long life with reasonably good quality, but even with modern treatment the disease still causes serious morbidity and increased mortality [2,3,4,5]. Most research has focused on the consequences of the lost beta-cell function, trying to improve the care of T1D, enhance the quality of life, avoid complications and reduce mortality. Therefore, whereas treatment guidelines and goals for blood glucose and HbA1c exist, there are no clear aims and do not exist any guidelines for the preservation of beta-cell function [1,6]. To some extent, this may explain why we still have no accepted, good enough treatment of the disease process.

Although the aim should be to cure the disease, even limited residual beta-cell function facilitates treatment, and reduces mortality [7] and the risk of both acute [8] and late [9] complications. C-peptide, which reflects beta-cell function and endogenous insulin secretion, is, therefore, an important clinical endpoint. In addition, there is evidence to suggest that C-peptide is a hormone with its own important effects [10]. 

Autoimmune diabetes in experimental animal models has allowed important research on different disease mechanisms and resulted in a large number of different methods that stop or delay the disease process. Unfortunately, these methods cannot easily be transferred into clinical trials as the results differ from animals to humans [11] and hitherto do not work in humans. Another problem delaying progress is the rough classification of T1D. For decades, the paradigm has been that T1D is a single homogenous disease. Therefore, clinical trials often include patients aged from 4 to 45 years at diagnosis (common in TrialNet studies), at times even up to 60 years, with different ethnic backgrounds, and sometimes with very different clinical picture and course [6]. The clinical experience that the course of T1D is quite different among pre-school children, school children, adolescents, and adults has been disregarded. Patient groups lumped in the same basket, with various types of disease process but given the same treatment in clinical trials, may be one reason for the failure of intervention trials. Another reason is that the requirements for sufficient improvement to reach endpoints have been quite hard. When treatment of other diseases leads to a minor improvement (decrease in certain symptoms or signs) in 10–20% of patients, this has been regarded as progress, whereas treatment of T1D has been regarded as a failure if it was effective in “only” 30–40% of the patients or if improvement in residual beta-cell function was “only” 15–20% for some years. In T1D there is little tradition of stepwise improvement in the disease process, but rather a tradition of either success or failure.

## 2. Different Forms of Immune Interventions

Plasmapheresis was used in the first immune intervention trial as autoantiantibodies were supposed to have a part of the destructive process [12], whereas Cyclosporin some years later was regarded as the proof of concept [13]. The most efficient immune therapy has been antiCD-3 treatment [14,15,16,17], especially in patients <18 years of age at onset of T1D. A new Phase 3, randomized, double-blind, placebo-controlled, multinational, multicenter study (Protect) is now ongoing to confirm efficacy in patients with the most suitable inclusion criteria (NCT03875729). Whereas recent results also suggest some efficacy in delaying the onset of T1D [18], the mechanisms are unclear as no results on the effect on C-peptide/beta-cell function have been published [19]. Although this treatment has been shown to be reasonably safe so far, associated adverse events and risks are extra important when treating children and adolescents. Furthermore, anti-CD3 treatment is heavy both for the patient and society, usually entailing 12–14 days i.v. treatment repeated two times. Besides anti-CD3, TNF-alfa inhibitors [20], ATG (Anti Thymocyte Globulin) [21], Alefacept [22] and Rituximab [23] are examples of drugs that have displayed greater efficacy than placebos, even though the effect on beta-cell preservation has usually been transient and limited. A serious drawback is that several of these treatment regimens have been quite heavy for the patients and a burden on the healthcare system. Most importantly, they have sometimes caused adverse events and risks that neither the patients, their parents/caregivers, nor diabetes teams are willing to accept given that the average patient will experience a long life with good quality with the existing traditional treatment. The better the conventional treatment becomes through the introduction of technical devices such as insulin pumps and glucose sensors, the fewer side effects and risks of immunotherapy will be accepted in clinical practice.

## 3. Autoantigen Treatment

### 3.1. Possible Mechanisms

Arguments exist for and against more traditional immune modulation and autoantigen treatment [24]. It can be postulated that immunomodulation using autoantigen treatment is far less toxic and dangerous than heavier forms of immune suppression/modulation and can give a much more specific response to the immune system than broader unspecific therapies do [11,25] The aim of autoantigen treatment is to reduce or stop a destructive specific immune response. If such “inverse” vaccination would work, it might be of tremendous value in the treatment of autoimmune diseases. In allergic disease, immunotherapy is used to create tolerance against the allergens by exposing the immune system to a suitable dose of the antigen/allergen/s. Such treatment has become increasingly efficacious, and adverse events are rare. It also seems plausible to try to reduce or stop an autoimmune process in an analogous way by administering autoantigen/s that might influence the existing immune balance. In animal models, there is evidence that antigen-specific immunotherapy is useful [11,26,27]. Evidence for the induction of antigen-specific regulatory T cells (Tregs) has been obtained in several experimental systems [28,29,30,31,32]. In the majority of cases, one treatment, or a short series of treatments, was sufficient to protect the animal from disease for the rest of its life. It was also possible to reverse established disease at the time of its onset [25,29].

Immunomodulation followed by infusion of antigen-specific Tregs has been shown to control autoimmunity in the pancreatic islets [33]. It has also been possible to decrease destructive CD8+cells in the pancreas by giving plasmid-encoding proinsulin [34]. Thus, whereas different techniques are being developed in animal experiments, studies in experimental animals do not prove that the treatment works in humans. We need human trials in which we try to modulate the immune response by presenting antigen/s in such a way that the immune system shifts from a destructive process to tolerance [35].

Little is known about the mechanisms for reaching tolerance or stopping the destructive process. If self-reactive T cells directed against autoantigens cause diabetes, at least in some cases of T1D, a major question is why such self-reactive T cells occur. The thymus, where naïve and competent T lymphocytes are generated, has a central role in the development of immunological self-tolerance. The T cells should be educated to recognize and tolerate proteins [36,37]. There are at least two mechanisms for developing self-tolerance: firstly, clonal deletion of self-reactive T cells issued from the random recombination of relevant genes (negative selection); secondly, the generation of self-antigen-specific natural Tregs. Tregs should be able to inactivate the self-reactive T cells in the periphery that have escaped intra-thymic negative selection. Autoantigen treatment might lead to immune modulation through the development of tolerance against certain autoantigens and by influencing the development of tolerogenic dendritic cells [38].

### 3.2. Autoantigen Treatment in Other Autoimmune Diseases than T1D

Autoantigen treatment is neither a new approach [39] nor limited to T1D. Studies on variants of multiple sclerosis (MS) exist since the 1990s [40,41,42,43,44,45] using this type of treatment. Moreover, peptide therapy has been tried in the treatment of MS [46,47]. The treatment was successful enough to lead to Phase III trials [48], but then failed to show beneficial effects. Autoantigen treatment has also been tried in myasthenia gravis [49], in rheumatoid arthritis, in systemic lupus erythematosus, [50,51], and in celiac disease [52,53]. However, these diseases encounter the same problems as those met in T1D, i.e., selecting the correct autoantigen, identifying the optimal timing, dosing and route of administration and, not least, finding biomarkers for monitoring the therapy.

### 3.3. What Autoantigen to Use in T1D?

One problem in the autoantigen treatment of T1D is to choose the most relevant autoantigen/s to use as there are so many beta-cell-related autoantigens (Table 1) [54]. There are autoantibodies and T-cell reactivity against insulin, glutamic acid decarboxylase (GAD65), insulinoma-associated protein 2/tyrosine phosphatase (IA-2), zinc transporter 8 (ZnT8), and also against proinsulin, the B-chain of insulin and proinsulin peptides. Furthermore, many more autoantigens have been described in recent years [54,55]. Hybrid insulin peptides consist of peptide fragments derived from both insulin and other insulin-secretory granule proteins which are fused together to form a hybrid peptide [56]. Furthermore, there are neoepitopes because of transformed antigens resulting from modifications that arise during antigen processing and presentation, and because of mechanisms such as peptide fusion and aberrant mRNA translation [57,58]. The cause may sometimes be endoplasmatic stress [59], which might increase abnormal post-translational modification of beta-cell proteins. In our own studies based on All Babies in Southeast Sweden (ABIS), we have found antibodies against oxidative posttranslationally modified insulin (oxPTM-insulin) [60], which predict the development of T1D [61]. Against such neoepitopes, both humoral and cell-mediated immunity is found [62]. Neoepites can probably be formed based on different proteins that are not only based on insulin and/or proinsulin, but also on islet amyloid polypeptide (IAPP) and its precursor, as well as other beta-cell proteins, and there are new findings coming. Thus, a new hybrid cell, called the X cell or dual expresser (DE) cell was recently found to have a receptor that expresses a new potent T cell autoantigen [63].

It is possible that not all neoepitopes can be identified in blood, as some may exist only in the beta-cells. This makes it even more difficult to select antigens for autoantigen treatment. Although it might be natural for the immune system to react against abnormal proteins/new neoepitopes, central tolerance may not be relevant to this process.

Thus, the selection of autoantigen/s for treatment is difficult. One criterion may be how the antigen fits the HLA type and thereby is presented to the immune system; another criterion may be to what extent the immune system evidently reacts against that autoantigen. In our own studies using GAD-alum treatment (see below) one inclusion criterion has been that the patient should have a certain number of antibodies against GAD65 (GADA). However, in recent analyses based on several studies we have preliminary data suggesting that HLA type should be one of the baseline parameters because the efficacy of GAD-alum treatment seems to be best in patients with HLA DR3/DQ2 but without HLA DR4/DQ8 [64].

### 3.4. Peptides in Autoantigen Treatment

Autoantigen therapies can either use the full autoantigen proteins or peptides from these antigens [65]. T1D should be a very suitable autoimmune disease for autoantigen or peptide therapy. Autoantibodies to antigens can be detected both in patients at the onset of T1D but also in individuals who are at high risk of getting the disease [66]. As antigen-specific T cells can be detected from the blood of patients and high-risk individuals [67], autoantigen/peptide therapy may be especially interesting in the future in the prevention of T1D [68]. However, whereas insulin given to prevent T1D did not work [69], it was found that oral insulin delayed onset of T1D in a subgroup of patients with high insulin autoantibodies (IAA) [70]. In experimental animals, intranasal insulin may prevent autoimmune diabetes [71], but intranasal insulin did not prevent T1D in a human clinical trial [72]. However, the possible efficacy of oral insulin has stimulated further studies to use peroral insulin for the prevention of T1D, and the PrePoint trial showed that such treatment of newborns with high genetic risk is safe and feasible [73]. In recent years a large trial has started in an effort to prevent T1D by treating newborns with high genetic risk of T1D, the GPPAD-POInT (Global Platform of Autoimmune Diabetes—Primary Oral Insulin Trial) (Clinical Trials Gov NCT03364868) in which 1040 individuals are planned to be enrolled at the age of 4–7 months of age and treated until 3 years of age. They are given oral insulin in a dose-escalation scheme. Primary outcomes are the development of multiple diabetes-related autoantibodies and the development of diabetes. GAD-alum has also been used in a small prevention trial. The treatment was safe and gave no adverse events, but no efficacy was found [74]. Peptide immunotherapy has been tried to restore immune homeostasis via the expansion of Tregs and the deletion and/or anergy of the pathogenic T-cell population [75]. In a clinical study in T1D patients, an altered peptide ligand (APL) derived from the insulin B9-23 epitope (NBI-6024) was administered and showed some initial positive results [65]. An antigen-specific T-cell response from a predominantly IFNγ response to a more Th2 response seemed to occur. However, no clinical benefit was shown in a subsequent clinical study in adult and adolescent patients with new-onset T1D to assess the effect of repeated administrations of NBI-6024 on residual beta-cell function measured by C-peptide [76].

In parallel to the clinical development of NBI-6024, a natural peptide sequence derived from proinsulin was evaluated. In a Phase 1 safety study in patients with long-standing diabetes and low C-peptide levels (<200 pmol/L), three monthly intra-dermal injections of 10 versus 100 µg of the C19-A3 peptide were compared [77]. The peptide seemed to be tolerable and safe. In addition, similar to the initial NBI-6024 study, there was a trend toward modulation of the antigen-specific T-cell response with an increased frequency of IL-10-producing cells in the low-dose treatment group. A second clinical study with a single proinsulin peptide was performed in newly diagnosed patients [78]. In this study, monthly versus fortnightly intra-dermal injections of 10 µg of peptide were compared over a period of 6 months. The peptide seemed to be well-tolerated and safe. There was evidence in some patients on the active treatment that the rate of decline in secreted C-peptide slowed down, and daily insulin use and HbA1c stabilized. However, it should be noted that in randomizing the three groups, the placebo patients had the more severe disease at baseline (higher HbA1c, higher insulin requirement and lower C-peptide) than had the patients in the two groups with active treatment. This makes it difficult to draw clear conclusions. In another randomized, four-arm, placebo-controlled, dose-ranging Phase 2 trial, repeated subcutaneous injections of an altered peptide ligand, NBI-6024, in an effort to inhibit autoreactive T-cells had no effect on preserving beta-cell function in patients with recent-onset T1D [76].

The clinical studies described so far have focused on either a single peptide to modulate the pathogenic T-cell response or multiple peptides from the same antigen. A different approach, using multiple peptides from two different autoantigens linked to the pathogenesis of T1D, is being evaluated as a treatment for new-onset T1D patients in a study called Multiple Islet Peptide Administration in type 1 diabetes (MultiPepT1De (ClinicalTrials.gov: NCT02620332)). This Phase Ib tolerability and safety study evaluating monthly intra-dermal injections of a cocktail of multiple peptides should provide new insights when data are published.

Selecting the optimal dose of autoantigen and the optimal dosing interval will be crucial. A problem will be to study the kinetics of antigens and distribution in the body. Better immunological tools are needed to track the antigen-specific T cells. 

### 3.5. GAD as Autoantigen

As mentioned above, the first immune intervention to be used in newly diagnosed T1D was plasmapheresis [12]. Those studies led to the discovery of a new diabetes-related antigen, with an estimated molecular weight of 64kD [79], which later was shown to be glutamic acid decarboxylase (GAD), actually with a molecular weight of 65kD. The GAD enzyme transforms glutamate to GABA (gamma-aminobutyric acid) and has a very important role in the central nervous system. Although GABA may regulate hormone release in the pancreas and/or work as a paracrine signaling molecule between the endocrine cells of the islets [80], the function of GAD in pancreatic islets is still not completely understood, nor is its role in the pathogenesis of T1D. In any case, GAD is a major autoantigen in T1D. Auto-antibodies toward GAD predict the development of T1D and are common in patients with T1D, and administration of GAD65 can prevent autoimmune destruction of pancreatic beta-cells in experimental animals [81,82,83,84]. This led to efforts to use this autoantigen in the treatment of humans. Thus, in spite of the challenges, we decided many years ago to try autoantigen treatment using GAD-alum, even though the mechanisms of its action were largely unknown.

GAD-alum was developed and, after necessary safety investigations, was used in a Phase 1 trial in LADA patients with promising results [85,86]. In the dose-finding Study 4, 20, 100 and 500 µg were tried, with 20 µg sc appearing to be as efficacious as 100 µg. This was followed by a Phase II clinical trial in Sweden including 70 recently diagnosed T1D children and adolescents in a randomized, double-blind, placebo-controlled multicenter study [87]. Twenty µg GAD-alum was given sc twice at one-month intervals. The treatment was very well tolerated without any serious treatment-related adverse events as yet after more than four years of follow-up. The primary endpoint (change in fasting C-peptide after 15 months), which was chosen based on a previous LADA trial, failed. However, there was a significant preservation of fasting C-peptide after 30 months (p = 0.045) as well as efficacy with respect to the C-peptide/plasma glucose ratio (p = 0.02). Stimulated C-peptide secretion, as measured by area under the curve (AUC) during a Mixed Meal Tolerance Test (MMTT) decreased less in the GAD-alum group than in the placebo group, after both 15 months (p = 0.01) and 30 months (p = 0.04) [88]. This difference remained significant after adjusting for differences in duration of diabetes, age, gender, and baseline GAD autoantibody (GADA) levels. 

The efficacy of the treatment was significantly influenced by the duration of diabetes when the treatment was given. Thus, in patients treated within 6 months of diagnosis, preservation of both fasting and stimulated C-peptide secretion (AUC) was significantly better in the group treated with GAD-alum than in the placebo group after 30 months (fasting p = 0.03 and stimulated p = 0.04), whereas no difference was observed when the patients had a duration of diabetes of >6 months. In patients with <6 months’ duration of diabetes at treatment, the effect on C-peptide preservation remained after more than 4 years [89]. Although preservation of C-peptide/residual insulin secretion has important clinical effects, insulin requirement, HbA1c and plasma glucose increased in both treatment groups over the course of the study. HbA1c did not differ between the groups, in accordance with the therapeutic target to keep HbA1c as near normal as possible in both treatment groups. 

The positive results of the first Phase II trial could not be confirmed in another randomized, double-blind, placebo-controlled Phase II trial [90]. In this study, with a different regimen, 145 patients were randomized in a double-blind, placebo-controlled study into three arms: one with sc injections of 20 µg GAD-alum at baseline, 4 weeks and 8 weeks; another with sc injections of 20 µg GAD-alum at baseline and 4 weeks, and placebo at 8 weeks; and a third with placebo at all time points. This study showed no significant preservation of C-peptide. A possible explanation for this failure may have been the inclusion criteria. Patients with a wide age range of 3–45 years were included in spite of the suspected heterogeneity of the disease.

Based on the positive Phase II trial [87] a European Phase III trial was performed [91] on 334 patients aged 10–20 years, with diabetes duration <3 months at screening, fasting C-peptide >0.1 nmol/L and positive GADA. In this study, the two arms of the Swedish Phase II study (placebo and 20 µg of GAD-alum, respectively) with a 30-day interval, were the same. However, in a third arm patients received 20 µg of GAD-alum (Diamyd) sc after 90 and 270 days. The patients in the other arms received placebo injections at these time points. The hypothesis was that additional booster doses of GAD-alum might improve efficacy. The study failed [92]. Preservation of C-peptide AUC was 16% greater in the actively treated patients than in those who were given a placebo (p = 0.10), and the difference in fasting C-peptide did not reach significance (p = 0.07). However, in several prespecified subgroups the efficacy was 30–60%, statistically and clinically significant. Efficacy was significant in boys; however, in line with tradition in T1D research, efficacy in boys alone was not considered good enough. Efficacy was significant in non-Nordic countries, leading us to surmise that the failure in Sweden and Finland was a result of the study coinciding with the H1N1 (swine flu) vaccination. The preservation of C-peptide was better the longer the time interval between GAD-alum treatment and the H1N1 vaccination was [93]. Moreover, efficacy was statistically significant in the rather few Swedish patients who were never given the H1N1 vaccination [94]. The results also seemed to be better in patients with high and moderate HLA risk and high GADA concentrations, but less good in patients with low HLA risk and low GADA. All these results seen in different subgroups illustrate the importance of better classification of T1D at diagnosis and knowledge about the disease process so as to define which treatment a patient should have. However, limited practical conclusions have been drawn so far.

**Figure 1:**
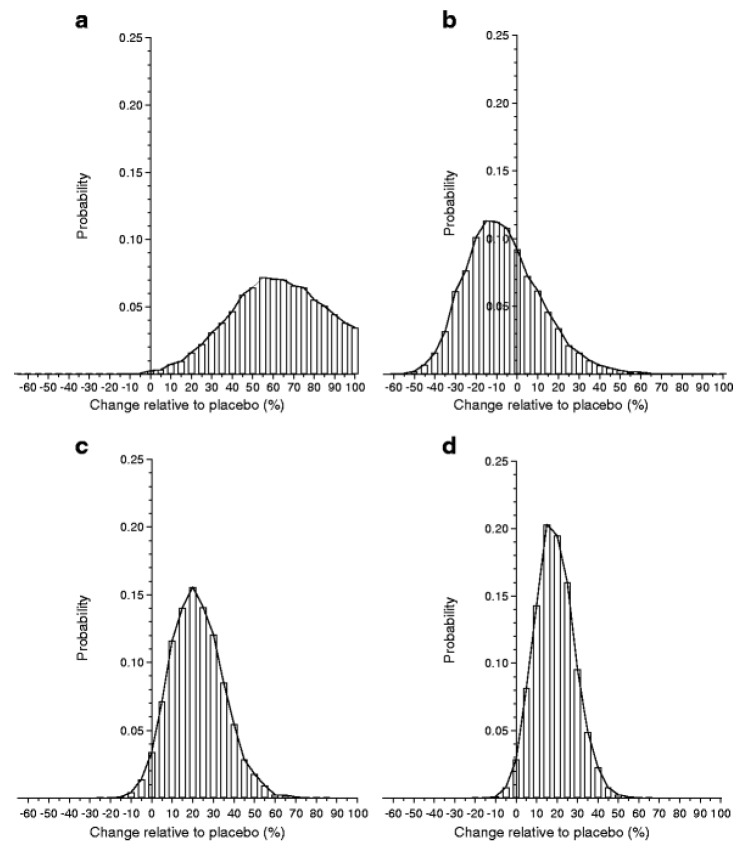
From Ref. [94] PPBE from the two-dose glutamic acid decarboxylase (GAD) vaccine. Histograms of the results from Bayes analysis of the effect of two-dose GAD treatment relative to placebo. Histogram bars are centered at 5% units representing a 5% increase (if positive) or decrease (if negative) in C-peptide change in treated individuals compared with placebo. The width of each bar represents a range of biological effects (change) of ±2.5% of the bar center. The height of each bar corresponds to the probability of that range of effect. The sum of the areas of the bars above zero gives the PPBE: (**a**) Ludvigsson (2008) [88], 99.8%; (**b**) Wherrett (2011) [91], 32.1%; (**c**) Ludvigsson (2012) [92], 96.6%; (**d**) meta-analysis, 98.0%.

Although previous studies have given diverging results, a meta-analysis suggests that the GAD-alum treatment is efficacious [95]. Thus, the probability of efficacy in the European Phase III trial was >97%, although that study did not meet the primary endpoint (Figure 1). This finding supports the need for further studies to try to improve efficacy, not least as GAD-alum given sc is extremely easy both for the patients and the healthcare system. A few extra injections at a month’s interval seem negligible for patients used to taking several injections every day. Besides studies in adults, GAD-alum (Diamyd) has been used in >250 children and teenagers, with a follow-up for several years showing no treatment-related serious adverse events and very mild, transient adverse events, i.e., mainly mild irritation at the injection site. Thus GAD-alum (Diamyd) given sc in the usual dose of 20 µg × 2–4 would appear to be safe. Even higher doses of up to 500 µg in a few patients have shown no adverse events. Theoretical risks of GAD-alum, such as acceleration of the autoimmune process, undesirable effects on the immune system and neurological disease, have been thoroughly evaluated. All clinical studies performed with GAD-alum (Diamyd) to date indicate a favorable safety profile, and no neurological concerns have been raised. Nevertheless, it remains possible, but unlikely, that side effects may occur in future clinical trials.

### 3.6. Different Routes for Administration of Autoantigen.

In treating the beta-cell disease process of T1D autoantigen, the autoantigen has been given either orally, intranasally or subcutaneously in an attempt to prevent T1D och preserve residual beta-cell function. In most studies mentioned above, the autoantigens have been given subcutaneously, but other routes have also been tried. Thus, insulin was given per os in the Diabetes Prevention Trial [69]. An initial inclusion criterion was insulin autoantibodies (IAA) of more than 80 U. However, as these individuals were difficult to find, the criterion was changed during the course of the study to IAA more than 40 U. On evaluation, the endpoint was not reached. Insulin per os did not delay the onset of T1D [69]. It was, however, found that in the initial group with IAA >80 U. there was a delay in T1D [70]. This led to another large prevention trial with insulin given per os, which showed some effect [96]. This has been followed by further studies with that route of administration of the autoantigen to test giving oral insulin to very young children to prevent T1D [97]. The encouraging results with respect to safety and feasibility have led to a large ongoing trial [98,99].

Oral insulin has also been tried in newly-diagnosed T1D but without effect [100,101]. A problem with oral administration is the degradation of proteins when they pass the gastro-intestinal tract. To overcome this problem, several different techniques have been used with promising results, at least in animal studies. The *Lactococcus lactis* bacteria has been used as a vehicle for different autoantigens [102,103,104], and autoimmune diabetes has been reversed in NOD mice [105]. Other vehicles used include attenuated *salmonella* [106] and *vaccinia* virus [107,108]. Antigens bioencapsulated in plant cells on oral delivery conferred immunity [109], and autoantigen-loaded phosphatidylserine liposomes may stop autoimmunity in experimental animals [110].

Beside oral administration, intranasal administration has been tried. This route might more easily present the antigen for dendritic cells and thereby increase modulation of the immune system [71,111]. Studies have therefore attempted to prevent T1D by giving insulin intranasally, but with no effect [72,73]. 

With any type of administration route, the autoantigen is supposed to be taken up by antigen-presenting cells, which should then move to the lymphatic tissue where they present the antigen to immune T cells. In the treatment of allergies, the traditional administration of allergens by sc injections requires years of repeated injections. Therefore, more efficient ways of administration have been evaluated. In animals, direct intra-lymphatic injections of antigen induce a strong T-cell response [112,113,114,115]. In treating allergy, clinical studies suggest that administration of the allergen directly into lymph nodes is more effective than other routes of administration. Lower doses and radically fewer numbers of treatments are needed, and there have been no treatment-related adverse events [116,117,118]. Senti et al. [116,117] found that three low-dose intra-lymphatic allergen administrations increased tolerance to nasal provocation with pollen within 4 months (p < 0.001). Tolerance was long-lasting and equivalent to that achievable after standard sc long-lasting immunotherapy. Intra-lymphatic immunotherapy ameliorated hay fever symptoms (p < 0.001), reduced skin prick test reactivity (p < 0.001), decreased specific serum IgE (p < 0.001), and still caused fewer adverse events than sc immunotherapy (p = 0.001). This therapy was regarded as less painful than venous puncture (p = 0.018), which also enhanced compliance (p < 0.001).

### 3.7. Intra-lymphatic Administration of Autoantigen

Intra-lymphatic autoantigen therapy has to my knowledge not been described either in animal experiments or in human autoimmune disease, including T1D. Knowing the difficulties of drawing valid conclusions from results in NOD mice we decided to test intra-lymphatic autoantigen administration directly into humans. To get the best effect on the immune process going on in the pancreas, it seems reasonable to give autoantigen as close to it as possible, yet in a way that is practical for the patient and for healthcare staff. Inguinal lymph nodes are situated close to the pancreas and are readily accessible in patients using an ultra-sound needle guide. The pain associated with intra-lymph-node injections has been rated as below that of a venous puncture. We decided to use GAD-alum for intra-lymphatic administration into inguinal lymph nodes in an open-label first-in-human pilot trial (DIAGNODE-1 (trial in type 1 diabetes giving GAD into lymph nodes; ClinicalTrials.gov IdentifierNCT02352974)). As previous studies had indicated that 20 µg GAD-alum sc given two times in a prime-and-boost regimen has a positive effect on C-peptide preservation, and allergy studies had shown that intra-lymphatic administration can achieve adequate efficacy with 3 injections of low allergen doses, a very low dose of 4 µg GAD-alum was injected directly into an inguinal lymph node three times with one-month intervals. The research ethics committee at Linköping University, Sweden (Dnr 2014/153-31) and the Swedish Medical Products Agency, Uppsala, Sweden (Dnr 5.1-2014-54385) initially allowed the inclusion of six adult patients aged 20–22 years with a diabetes duration of <6 months after they had given their written informed consent. Other inclusion criteria were GADA positivity (GADA>63 U but < 50 000 U) with fasting C-peptide >0.12 nmol/L (0.36 ng/mL). An ultra-sound needle guide was used to give the patients three injections of 4 μg of GAD-alum into an inguinal lymph node at one-month intervals, on days 30, 60 and 90 of the trial. In addition, they received a vitamin D (calciferol) supplement in oral solution (2000 U/d) on days 1–120 (see rationale below). 

Beta-cell function was estimated with mixed-meal tolerance tests at baseline and then after 6, 15 and 30 months. Immune function was assessed by cell proliferation assays, flow cytometry, determination of cytokines (Bio-Rad, Luminex) and GAD autoantibodies including sub-classes. The first results looked very encouraging [119,120].

### 3.8. Adjuvant Vitamin D 

The completely novel approach of administering auto-antigens intra-lympatically may mean improved efficacy, but there are reasons to believe that combination therapy should be tried in an effort to preserve beta-cell function in T1D [6,121,122,123]. Epidemiological studies suggest a link between vitamin D deficiency and an increased incidence of T1D, showing that higher vitamin D3 at birth may protect from T1D later in life [124,125]. A meta-analysis supports these conclusions [126]. Others report lower serum levels of 1α,25 -dihydroxyvitamin D3 [1,25(OH)2D3, calcitriol] in patients with recently diagnosed T1D than in healthy control subjects [127]. One study has shown a lower risk of developing T1D in individuals with pre-diagnostic vitamin D levels above 100 nmol/L compared with levels below 75 nmol/L [128]. Evidence suggests that vitamin D affects beta-cells and makes them more resistant to cellular stress [129]. Certain results even suggest that vitamin D may improve insulin sensitivity [130], which in turn may decrease beta-cell stress. Nevertheless, vitamin D given as a single treatment has not been efficacious enough to preserve beta-cell function [131,132]. On the other hand, 1,25(OH)2D3 seems to modulate dendritic cell maturation in vitro and in vivo and to facilitate a shift from a Th1 to a Th2 immune response [133,134,135,136,137]. Altogether, this supports the importance of sufficient vitamin D levels for a tolerogenic response to immunotherapy with beta-cell antigens. Therefore, there is a reason to ensure that vitamin D levels are adequate in patients receiving autoantigen treatment. Therefore, intra-lymphatic GAD immunotherapy was combined with oral vitamin D.

### 3.9. Experience from Intra-Lymphatic GAD-alum Treatment

Intra-lymphatic GAD-alum treatment was easy to administer, with no treatment-related adverse events except for a mild transient reaction at the injection site. The results were encouraging regarding beta-cell preservation and immunological response [119,120]. With this background in adult patients the Swedish Medical Product Agency and the research ethics committee at Linköping University approved an amendment of the protocol to allow patients from age 12 to be included. In total 12 patients were included (4 females, 8 males; 12, 6–23, 1-year-old). After 15 months, the results are encouraging (to be published). The study has no statistical power to prove efficacy. However, small pilot studies can be useful in gaining insight before conducting large trials [25,138]. Therefore, we have even taken an additional step to gain greater insight and have given the first three adult patients a 4th booster GAD-alum intra-nodal injection after 30 months. The follow-up is too short to draw conclusions about the results, but we have seen no adverse events to date; if anything, we have an initial improvement of beta-cell function. 

With the encouraging results from the pilot DIAGNODE-1 trial, it was decided to conduct a randomized double-blind, placebo-controlled Phase II trial with the same design as the DIAGNODE-1 trial. The study is a 2-arm, randomized, double-blind, placebo-controlled study in 106 GADA positive T1D patients aged ≥12 and <25 years old, diagnosed within 6 months prior to screening with fasting C-peptide levels ≥0.12 nmol/L (0.36 ng/mL). 

The main goal is to find a reasonably safe and tolerable treatment for young and adult patients with T1D that can preserve residual insulin secretion, improve quality of life (QoL), and reduce the risk of short-term and long-term complications.

The primary objective is to evaluate the efficacy of GAD-alum (Diamyd), administered into lymph nodes, with oral vitamin D supplementation up to adequate levels, compared with placebo, in terms of preserving endogenous insulin secretion as measured by C-peptide.

The secondary objectives are to compare GAD-alum, administered into lymph nodes in combination with an oral vitamin D regimen and placebo treatment with respect to the effects on diabetes status, treatment safety, immune system and quality of life (QoL). Endpoints are shown in Box 1 and Box 2.

Box 1 **Primary and secondary endpoints in a clinical trial (DIAGNODE-2) evaluating the efficacy of intra-lymphatic autoantigen treatment of type 1 diabetes.**

The primary endpoint

- Change in C-peptide (Area Under the Curve [AUC mean 0–120 min) during a mixed-meal tolerance test (MMTT) between baseline to 15 months.

The secondary endpoints to evaluate diabetic status:

- Proportion of patients with a stimulated maximum C-peptide level above 0.2 nmol/L (0.6 ng/mL) at 15 months.
- Proportion of patients with a stimulated 90min C-peptide level above 0.2 nmol/L (0.6 ng/mL) at 15 months.
- Change in maximum C-peptide during MMTT between baseline and 15 months.
- C-peptide measured at 30, 60, 90, and 120 min during MMTT at 15 months.
- Change in fasting C-peptide between baseline and 15 months.
- Change in HbA1c between baseline and 15 months.
- Change in daily exogenous insulin consumption between baseline and 15 months.
- Change in insulin-dose-adjusted HbA1c (IDAA1c) between baseline and 15 months.
- Proportion of patients with by IDAA1c ≤ 9 at 15 months.
- Number of self-reported episodes of severe hypoglycemia (Severe hypoglycemia defined as needing help from others and/or seizures and/or unconscious) between baseline and 15 months.
- Change in rate of hypoglycemic events between baseline and 15 months.
- Number of patients having at least 1 severe hypoglycemic event between baseline and 15 months.
- Change in glycemic variability/fluctuations (evaluated from data from continuous glucose monitoring FreeStyle LibrePro, Flash Glucose Monitoring [FGM]) over 14-day period between screening and 15 months.

The secondary endpoints to evaluate safety:

- CInjection site reactions.
- Occurrence of adverse evernts (AEs).
- Laboratory measurements (hematology and clinical chemistry).
- Urine analysis (microalbuminuria, creatinine).
- Physical examinations, including neurological assessments.
- GAD65A titer.
- Vital signs (blood pressure).

Box 2 **Secondary endpoints in a clinical trial (DIAGNODE-2) evaluating the efficacy of intra-lymphatic autoantigen treatment of type 1 diabetes.**

The secondary endpoints to evaluate the influence on the immune system:

- Concentrations of serum autoantibodies toward GAD65 and IA 2.
- Concentrations of serum autoantibody isotypes toward GAD65.
- Total serum Immunoglobulin E (IgE).
- Secretion of interleukin (IL)-1, IL-2, IL-5, IL-13, IL-10, IL-17, interferon (IFN)γ, and tumor necrosis factor (TNF)α by peripheral blood mononuclear cells (PBMCs) on stimulation with GAD65.
- Proliferation of PBMCs on stimulation with GAD65.
- Flow cytometric analysis of PBMC subsets.
- Further exploratory immunological characterization.

The secondary endpoints of quality of life (QoL):

- Change in QoL as measured by questionnaire EQ-5D-5L between baseline and month 15.
- Quality-adjusted life years (QALYs) based on the EQ-5D-5L questionnaire.

## 4. Challenges and Perspectives

The incidence of T1D is increasing worldwide [138,139,140]. The etiology is unknown, but genetic [141] and environmental factors [142] play an important role. To date, no method is known to prevent the disease. Despite better insulins and tremendous progress in techniques to facilitate treatment, the disease is still very serious, with a high risk of complications [5], increased mortality in spite of improved metabolic control [4] and with many years reduced life expectancy [5]. Preserved beta-cell function is of the utmost importance. However, as yet no method has shown long-lasting preservation of residual beta-cell function in T1D, at least not without quite severe adverse events and risks. 

Single therapies seem less likely to work. Autoantigen treatment in combination therapies may be a way forward. Another step forward is to better classify type 1 diabetes and already the presymptomatic stages [143]. Several unsolved questions remain regarding the selection of autoantigen dose, administration route and markers to follow efficacy. So far, most autoantigen therapies have shown limited or no efficacy. With the large number of autoantigens involved in T1D, it is plausible that different autoantigens may fit different patient groups depending on HLA and immune pattern at disease onset. Furthermore, we need to learn more about how, when and in what doses the autoantigen should be administered. It is also conceivable that efficacy will be improved by combining autoantigen treatment with other immunomodulatory regimens as well as with protective agents [120].

There is a large and growing number of autoantigens that can be used in the treatment of T1D. Glutamic acid decarbolyxylase (GAD) is one of the most studied autoantigens in human clinical studies. GAD has been combined with alum hydroxide to form GAD-alum (Diamyd), and treatment with this agent has been shown to be easy to use, tolerable and safe. Although the results from different studies have varied, and primary endpoints not been met, it seems probable that treatment with 20 µg sc GAD-alum (Diamyd) twice at one-month intervals is efficacious in children and teenagers with recent-onset T1D. Bayesian statistics show a very high probability of efficacy, justifying further efforts to improve this. Analyses of a large number of patients treated in several published GAD-alum Phase II/III trials suggest that the response is best in patients positive for HLA DR3/DQ2, but negative for DR4/DQ8. These individuals often have higher GADA concentrations but lower IA-2A. Alone or in combinations, autoantigen therapy will be a very attractive form of treatment and is likely to be desired by patients as it is easy, tolerable, without risks and side effects, and popular among physicians and other healthcare staff as it is easy to use without hospitalization. If new administration forms such as intra-lymphatic treatment show promise, this might lead to efforts to use a similar approach in several auto-immune diseases.

## Figures and Tables

**Table 1:** Beta-cell-related autoantigens in type 1 diabetes.

Commonly Discussed “Old”Autoantigens	More Recently Described Antigens/Neoepitopes
Insulin	Tetraspanin-7, Glima38
Proinsulin	Islet Amyloid Polypeptide (IAPP)
Insulin B-chain	Human IAPP Precursor Protein (ppIAPP)
Proinsulin peptides eg C19-A3	ChgA (Chromogranin A)
Glutamic Acid Decarboxylase (GAD65)	IGRP Islet-Specific Glucose-6-Phosphatase Catalytic Subunit-Related Protein
Insulinoma-Associated Antigen; (IA-2)	Insulin-Gene Enhancer Protein Isl-1
Tyrosine Phosphatase (IA-2)	Peripherin
Zinc Transporter 8-Antigen.	P4Hb (Prolyl 4-Hydroxylase Subunit Beta)
Islet Cell Antigen (ICA); a mixture	P4Hb (Prolyl 4-Hydroxylase Subunit Beta)
	GRP78 (Glucose-Regulated Protein 78)
	Urocortin-3
	Oxidative Post-Translational Modifications (oxPTM)-Insulin
